# Quinine in Cholera Infantum

**Published:** 1851-10

**Authors:** G. W. Booth

**Affiliations:** Hardin county, Tenn.


*Quinine in Cholera Infantum.* By G. W. Booth, M. D., of Hardin county, Tenn.—I have for sometime intended to call the attention of my professional brethren to the quinine mode of treating Cholera Infantum. I have practised in Mississippi and Tennessee for several years—regions where that disease annually makes its visitations. I have had an opportunity of seeing much of it, and treating a great number of cases. I believe its remote cause to be identical with that of our common autumnal fevers.

It prevails at the same period of the year, and in the same locations, and I have found it amenable to the same treatment, aided by the usual remedies for the local complications. There are in all the cases that have fallen under my notice distinct remissions, if not intermissions. During this subsidence of the symptoms, I give quinine liberally, and, in fact, in many cases I give it, regardless of fever, throughout the disease. I can confidently recommend this mode of treatment as preeminently successful, after testing it for many years. I have seen many cases recover under the use of the sulphate, that I should have despaired of without its aid. I write this simply to get you to call the attention of the profession to the use of quinine in this disease. I have never written an article for the medical press; and this is not intended for the public eye.* I do not expect that any, or at least many, will adopt any mode of treatment differing from what is usual, on the recommendation of an obscure practitioner—one entirely unknown to fame. As this is the season for the prevalence of Cholera Infantum in your city, I request you to give it a full and fair trial; and if you find it successful, then send it abroad under the prestige of your Gazette. I use the quinine in every stage of the disease, in what would by many be considered large doses. Occasionally I defer its use for a short time, to relieve some of the most urgent symptoms—such as excessive gastric irritability, and cerebral affections.—*New York Med. Gaz.*

---

[*We insert it, nevertheless, in the hope that Dr. B. and others of what are called country practitioners will be encouraged to write for the medical press.—Ed.]

